# Phyllanthin from *Phyllanthus amarus* exerts neuroprotective effects against spinal cord injury in experimental rats

**DOI:** 10.1055/s-0045-1809408

**Published:** 2025-06-17

**Authors:** Juan He, Yang Cheng, Yuekun Yang, Zhaofeng Fan

**Affiliations:** 1 Sichuan University, West China Hospital, West China School of Nursing, Department of Neurosurgery, Chengdu Sichuan, China.

**Keywords:** Apoptosis, Inflammation, Phyllanthus, Spinal Cord Injuries

## Abstract

**Background:**

Spinal cord injury (SCI) results in changes in autonomic function, impacting an individual's movement, sensory perception, and overall quality of life. Phyllanthin, a lignan from *Phyllanthus amarus* , is known for its neuronal protective effects.

**Objective:**

To evaluate the potential of phyllanthin identified in *P. amarus* methanolic extract (PAME) against SCI in experimental rats.

**Methods:**

The lignan was identified in PAME using high-performance liquid chromatography (HPLC). Spinal cord injury was induced in Sprague-Dawley rats using the laminectomy clip compression method. Rats received either a vehicle (distilled water) or methylprednisolone (30 mg/kg) or PAME (50, 100, and 200 mg/kg) orally for 4 weeks after SCI. Behavioral, histological, and molecular parameters were assessed to evaluate the neuroprotective effects of phyllanthin.

**Results:**

During the HPLC analysis of PAME, phyllanthin was present at a retention time of 25.30 minutes with 75.22% weight per weight (w/w). The administration of standardized PAME (100 and 200 mg/kg) effectively ameliorated the alterations induced by SCI in thermal and mechano-tactile hyperalgesia, locomotor activity, and nerve conduction velocity ( *p* < 0.05 each). The SCI-induced elevation in spinal interleukins (ILs: IL-1β and IL-6) and tumor necrosis factor alpha (TNF-α) protein levels was also effectively ( *p* < 0.05) reduced by PAME. The PAME treatment markedly ( *p* < 0.05) ameliorated SCI-induced alterations in protein expressions of B-cell leukemia/lymphoma 2 (Bcl-2), Bcl2-associated X protein (Bax), and caspase-3 in the spinal cord. Aberrations, such as inflammatory infiltration, edema, congestion, and necrosis induced in the spinal cord, were also effectively reduced by the PAME treatment ( *p* < 0.05).

**Conclusion:**

Phyllanthin identified in *P. amarus* showed neuroprotective potential against SCI by moderating impairments in behavioral (allodynia, hyperalgesia, and nerve conduction velocity) parameters, elevated inflammatory mediators (IL-1β, IL-6, and TNF-α), and deactivating the apoptotic signaling (Bax/caspase-3) pathway.

## INTRODUCTION

Damage to the spinal cord, known as spinal cord injury (SCI), can occur due to traumatic events (including falls, accidents, injuries etc.), disease or infections (such as meningitis), ischemia, as well as other causes. It can be classified as temporary or permanent, and it is associated with alterations in motor, sensory, and autonomic functions that affect mobility, sensation, and overall quality of life. 1 Evidence 2 suggests that SCI is associated with significant treatment costs, which are higher in developed countries. The high costs of SCI are associated with emergency care, rehabilitation, and lifelong support. This injury required specialized medical interventions such as spinal surgery, ventilatory support, and physical therapy. A recent systematic review 2 documented the cost of care for SCI ranging from $290 to $612,590, with the cost of inpatient rehabilitation ranging from $19,360 to $443,040. Individuals with SCI may also experience complications such as pressure ulcers, urinary tract infections, respiratory issues, neuropathic pain etc., and managing these complications involves additional medical expenses. 1 Thus, access to comprehensive healthcare coverage, rehabilitation services, and support programs is essential to help mitigate the financial burden on individuals and families affected by SCI.

The pathophysiology of SCI involves complex mechanisms contributing to tissue damage and neurological dysfunction. 3 Primary injury encompasses immediate physical damage to the spinal cord and subsequent secondary injury, which consists of a cascade of events, including inflammation, ischemia, excitotoxicity, oxidative stress, and apoptosis, resulting in tissue damage and neurological deficits. 4 5 Inflammatory responses play a critical role during SCI, in which the activation of immune cells, including microglia and macrophages, causes the release of chemokines, cytokines, and reactive oxygen species. Excitotoxicity is another important mechanism of SCI, which is characterized by the excessive activation of glutamate receptors, contributing to neuronal death and tissue damage. Symptoms including paralysis or weakness, loss of sensation, altered reflexes, bowel and bladder dysfunction, respiratory compromise, and autonomic dysfunction clinically represent this cascade of events. Additionally, individuals with SCI are at risk of various complications, including deep vein thrombosis, pressure ulcers, spasticity, urinary tract infections, respiratory complications, neuropathic pain, and autonomic dysreflexia.

Phyllanthin is one of the lignans widely present in *Phyllanthus amarus* , *Phyllanthus urinaria* , and *Phyllanthus niruri* that exhibits antioxidant, antidiabetic, antiviral, hyperlipidemic, nephroprotective, hepatoprotective and anti-inflammatory potential. 6 7 The mechanisms supporting its various pharmacological properties involve the modulation of nuclear factor erythroid 2–related factor 2 (Nrf2), nuclear factor kappa B, cyclooxygenase-2, interleukin (ILs), and tumor necrosis factor alpha (TNF-α), B-cell leukemia/lymphoma 2 (Bcl2), Bcl2-associated X protein (Bax), and caspase-3. 6 7 Nevertheless, the effectiveness of phyllanthin against SCI remains unexplored. Consequently, the present investigation aimed to assess the neuroprotective potential of phyllanthin identified in *P. amarus* methanolic extract (PAME) against SCI in experimental rats.

## METHODS

### Preparation and identification of phyllanthin in PAME

The procedure was performed following a previously-documented method. 8 In summary, 500 g of air-dried powder from *P. amarus* aerial parts were soaked and agitated in distilled methanol at room temperature for a week, then strained. The resulting liquid was then dehydrated in a tray dryer at 40 °C. The obtained semisolid PAME was combined with colloidal silicon dioxide and further dried in a vacuum tube dryer. The PAME underwent phytochemical analysis to determine its phyllanthin content using high-performance liquid chromatography (HPLC; Chemie-Erzeugnisse und Adsorptionstechnik AG). The analysis employed an HPLC system with a reversed-phase column 18 (RP C18; 5µ; 250 × 4.6 mm), a flow rate of 1.5 mL/minute, an injection volume of 20 μL, and the detector's wavelength of 230 nm. The mobile phase consisted of acetonitrile:buffer (40:60), with the buffer containing 0.136 g of potassium hydrogen phosphate and 0.5 mL of o-phosphoric acid. The autosampler was maintained at 10° C, and the system pressure was maintained at 1,000 psi.

### Induction of SCI and drug treatment schedule

The experimental protocol for the induction of SCI was approved by West China Hospital (under number 20240301191). Adult male Sprague-Dawley rats (weight: 180–220 g; age: 7–8 weeks, sourced from West China Hospital; maintained 24 ± 1 °C; normal light and dark cycle; relative humidity: 45 to 55%; and free access to feed and water) underwent SCI induction. The procedure involved single-level laminectomy at T10 using a temporary aneurysm clip technique. In this method, a consistent closing force is applied to the spinal cord for 60 seconds. 9 After recovery, the rats were divided into groups of 15 individuals each: sham, SCI control (both groups received 10 g/kg of distilled water), methylprednisolone (30 mg/kg in distilled water), *and P. amarus* (50, 100, and 200 mg/kg in distilled water). 8 Another set of normal animals was also maintained, which underwent surgery but were not submitted to SCI, and received 10 g/kg of distilled water. The rats received their respective treatments orally for 28 days.

### Outcome assessment

Behavioral test parameters, including mechano-tactile allodynia (Von Frey hair apparatus; IITC Life Science), thermal hyperalgesia (tail-flick test), locomotor activity (open field test; VJ Instruments), and motor nerve conduction velocity (MNCV) were recorded on days -2, 0, 7, 14, 21, and 28, as previously described. 10

The weights of various reproductive and urinary organs were measured, including the seminal vesicle, testis, urinary bladder, prostate gland, epididymis, and kidneys. Sperm count was assessed using a previously-described technique. 5 A spectrophotometer (UV-visible spectrophotometer, Jasco V-530) was employed to measure urine protein levels, using reagent kits in accordance with the manufacturer's protocol (Accurex Biomedical Pvt. Ltd.).

On the 29th day, the animals were anesthetized under ethereal anesthesia and then sacrificed through cervical dislocation. The injured or lesioned section of each rat's spinal cord dorsal horn was extracted and preserved at -80°C for subsequent analysis. Biochemical analyses were conducted to measure the levels of IL-1β, IL-6, and TNF-α using rat-specific enzyme-linked immunosorbent assay (ELISA) kits (Bethyl Laboratories Inc.) Western blot analysis was performed to assess the expression of Bax (E63, ab32503; Abcam Trading (Shanghai) Co. Ltd.), Bcl2 (EPR17509, ab182858), and caspase-1 (EPR19672, ab238979) protein according to a previously-reported method. 10 Additionally, a separate portion of the injured or lesioned dorsal horn from three rats in each group was fixed for histological examination. The extent of the SCI was evaluated using a light microscope (E200, Nikon Corporation) at a magnification of 40x, as per a previously-described scoring system as follows: score of 0 (when absent), score of 1 (when present in 0–25% of the examined myocardium), score of 2 2 (when present in 25–50% of the examined myocardium), score of 3 (when present in 50–75% of the examined myocardium), and score of 4 (when present in 75–100% of the examined myocardium). 11

### Statistical analysis

The results were expressed as mean and standard error of mean (SEM) values. The statistical analysis was conducted using the GraphPad Prism software (GraphPad Software, Inc.). For the results of the behavioral test, two-way repeated measures analysis of variance (ANOVA) was employed, followed by the Bonferroni's post-hoc test. Biochemical parameter data were evaluated using one-way ANOVA, with subsequent post-hoc analysis using Tukey's multiple range test. Statistical significance was established at *p* < 0.05 for all analyses.

## RESULTS

### Identification of Phyllanthin in PAME

**Figure 1:**
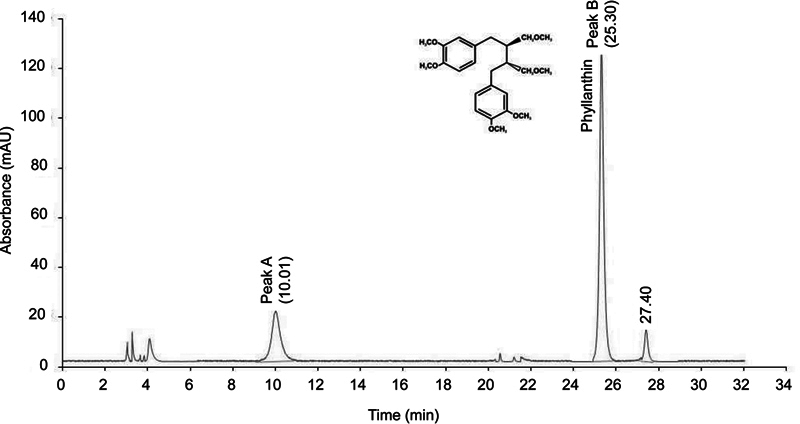
High-performance liquid chromatography (HPLC) fingerprint of the standardized *Phyllanthus amarus* extract reflecting a major peak of phyllanthin (peak B; retention time: 25.30 minutes).

The PAME showed the presence of flavonoids, lignans, alkaloids, tannins, steroids, and terpenoids. The percentage yield of the methanolic extract was of 75.22% w/w. The HPLC chromatogram showed a retention time of 25.30 minutes for phyllanthin ( Figure 1 ).

### Body weight, intakes (food and water), urine parameters (output and protein)

The SCI control group exhibited a significant ( *p* < 0.05) reduction in body weight and intake (food and water), along with an increase in urine parameters (output and protein) levels than the sham and normal rats. The administration of PAME (100 and 200 mg/kg) and methylprednisolone led to a significant ( *p* < 0.05) body weight gain and increase in intake while decreasing urine parameters compared to the SCI control group. Methylprednisolone demonstrated greater efficacy in mitigating SCI-induced changes in these parameters than the PAME (50 mg/kg) treatment. No significant ( *p* > 0.05) differences were observed between sham and normal rats regarding body weight, intake and urine parameters ( Table 1 ).

**Table 1:** Mean values for body weight, intake (food and water), urine parameters (volume and protein), sperm count, weights of the seminal vesicle, testis, urinary bladder, prostate gland, kidney, and epididymis

Parameter	Normal	Sham	SCI-control	MP (30 mg/kg)	PA (50 mg/kg)	PA (100 mg/kg)	PA (200 mg/kg)
Body weight (g)	226.50 ± 1.38	227.30 ± 1.98	168.20 ± 1.30 ^#,&^	218.20 ± 2.27* ^,$^	178.30 ± 2.12	192.50 ± 1.84* ^,$^	211.00 ± 1.75* ^,$^
Food intake (g)	23.33 ± 0.49	22.17 ± 0.4	11.00 ± 0.58 ^#,&^	19.67 ± 0.95* ^,$^	13.50 ± 0.43	15.50 ± 0.50* ^,$^	17.83 ± 0.87* ^,$^
Water intake (mL)	39.17 ± 0.95	43.17 ± 0.75	23.33 ± 1.05 ^#,&^	37.00 ± 0.97* ^,$^	25.50 ± 0.89	29.83 ± 1.08* ^,$^	34.67 ± 1.61* ^,$^
Volume of urine (mL) expressed in 24 hours	1.33 ± 0.21	1.83 ± 0.17	5.33 ± 0.21 ^#,&^	2.33 ± 0.21* ^,$^	4.50 ± 0.22	3.33 ± 0.21* ^,$^	2.67 ± 0.21* ^,$^
Urine protein (mg/mL)	1.39 ± 0.15	2.53 ± 0.23	6.84 ± 0.15 ^#,&^	3.41 ± 0.27* ^,$^	6.33 ± 0.19	4.47 ± 0.21* ^,$^	3.90 ± 0.12* ^,$^
Sperm count (million/mL)	53.67 ± 2.81	52.50 ± 2.68	28.50 ± 1.43 ^#,&^	46.33 ± 2.77* ^,$^	29.33 ± 1.91	36.50 ± 1.65* ^,$^	49.83 ± 2.80* ^,$^
Seminal vesicle weight (g)	2.47 ± 0.09	2.09 ± 0.08	1.09 ± 0.09 ^#,&^	2.12 ± 0.13* ^,$^	1.21 ± 0.11	1.72 ± 0.11* ^,$^	1.97 ± 0.11* ^,$^
Testis weight (g)	2.60 ± 0.11	2.28 ± 0.10	1.02 ± 0.09 ^#,&^	2.17 ± 0.10* ^,$^	1.24 ± 0.09	1.71 ± 0.09* ^,$^	1.99 ± 0.11* ^,$^
Urinary bladder weight (g)	0.42 ± 0.01	0.44 ± 0.01	1.18 ± 0.01 ^#,&^	0.59 ± 0.01* ^,$^	1.10 ± 0.02	0.91 ± 0.02* ^,$^	0.69 ± 0.02* ^,$^
Prostate gland weight (g)	0.46 ± 0.01	0.45 ± 0.01	0.27 ± 0.02 ^#,&^	0.42 ± 0.02* ^,$^	0.28 ± 0.01	0.33 ± 0.01* ^,$^	0.42 ± 0.01* ^,$^
Kidney weight (g)	126.80 ± 3.64	126.70 ± 2.51	103.20 ± 3.30 ^#,&^	125.20 ± 3.40* ^,$^	109.80 ± 3.83	112.00 ± 2.67* ^,$^	118.70 ± 4.52* ^,$^
Epididymis weight (g)	2.64 ± 0.06	2.56 ± 0.08	1.36 ± 0.04 ^#,&^	2.38 ± 0.10* ^,$^	1.53 ± 0.06	1.81 ± 0.06* ^,$^	2.08 ± 0.08* ^,$^

Abbreviations: MP, methylprednisolone; N, normal; PA, *Phyllanthus amarus* ; S, sham; SCI, spinal cord injury.

Notes: Results are expressed as mean and standard error of the mean ( *n* = 6) values. The statistical analysis was conducted using one-way analysis of variance (ANOVA) with the Tukey's post-hoc test for multiple comparisons. *p* < 0.05: *SCI-control; ^#^ sham; ^&^ normal; and ^$^ one another (methylprednisolone versus *P. amarus* ).

### Organ weights and sperm count

In the SCI control rats, significant reductions ( *p* < 0.05) were observed in the organ weights, including the seminal vesicle, testis, epididymis, prostate gland, and kidneys, while the urinary bladder weight showed a significant increase ( *p* < 0.05) compared to the normal and SCI control rats. Additionally, the SCI control rats exhibited a significant decrease ( *p* < 0.05) in sperm count relative to the normal and SCI control rats. The administration of PAME (100 and 200 mg/kg) and methylprednisolone significantly mitigated ( *p* < 0.05) SCI-induced changes in sperm count and organ weight compared to the SCI control rats. Moreover, a significant difference ( *p* < 0.05) was observed between the rats treated with PAME (50 mg/kg) and those treated with methylprednisolone in terms of their ability to alleviate SCI-induced alterations in sperm count and organ weight ( Table 1 ).

### Behavioral parameters

**Figure 2:**
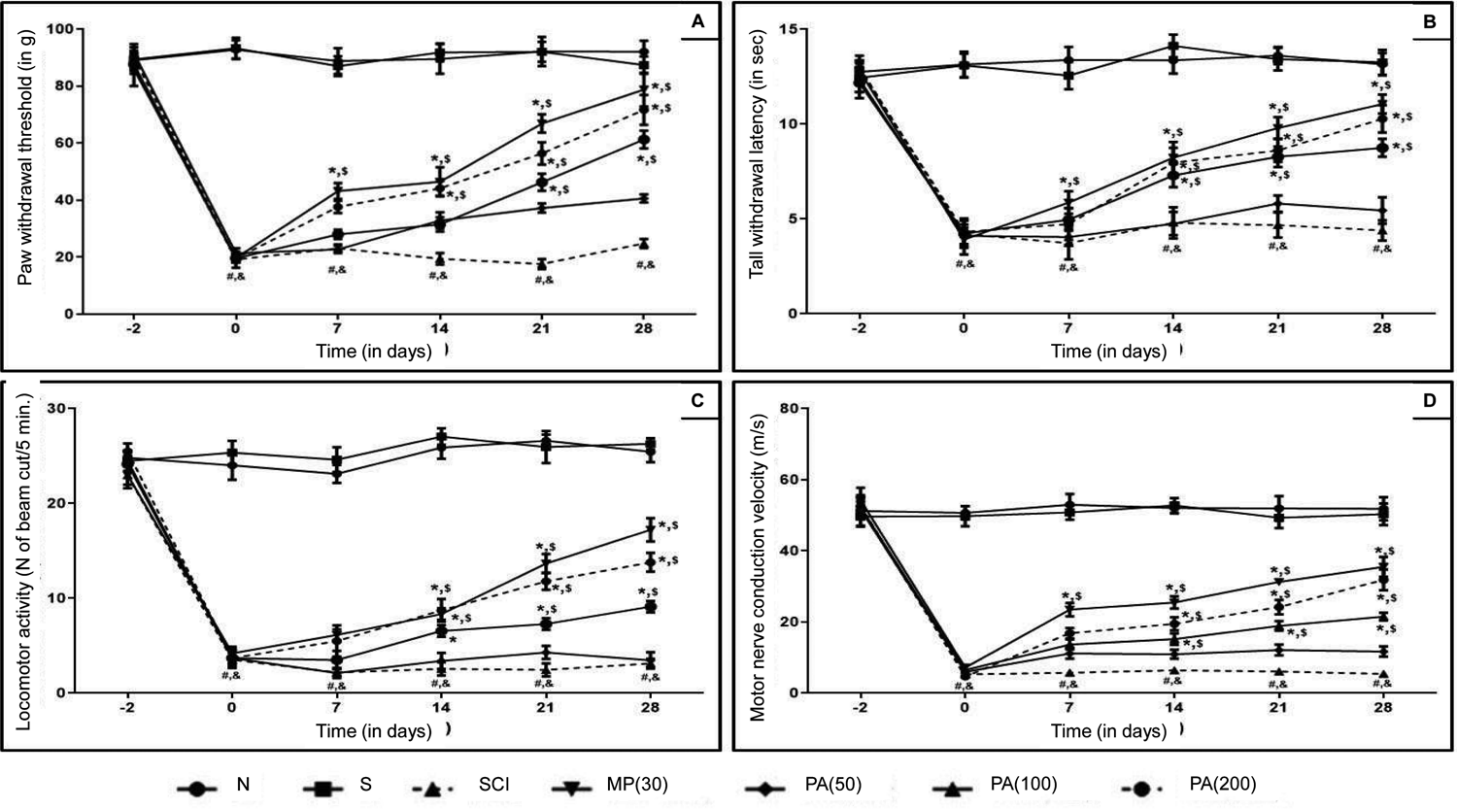
Abbreviations: MP, methylprednisolone; N, normal; PA, *Phyllanthus amarus* ; S, sham; SCI, spinal cord injury. Notes: Results are expressed as mean and standard error of the mean ( *n* = 6) values. The statistical analysis was conducted using one-way analysis of variance (ANOVA) with the Tukey's post-hoc test for multiple comparisons. *p* < 0.05: *SCI-control; ^#^ sham; ^&^ normal; and ^$^ one another (methylprednisolone versus *P. amarus* ). Mechanical allodynia in the von Frey hair test ( **A** ), thermal hyperalgesia in tail immersion test ( **B** ), locomotor activity in an open field ( **C** ), and motor nerve conduction velocity ( **D** ).

On day -2, there was no significant difference ( *p* > 0.05) in terms of thermal hyperalgesia, mechano-tactile, locomotor activity, and MNCV among the normal, sham, and SCI control rats. However, from days 0 to 28, a significant ( *p* < 0.05) decrease in thermal hyperalgesia, mechano-tactile, locomotor activity, and MNCV was observed in the SCI control rats compared to the sham and normal rats. The PAME (100 and 200 mg/kg) and methylprednisolone treatment demonstrated a significant ( *p* < 0.05) increase in thermal hyperalgesia, mechano-tactile, locomotor activity, and MNCV in comparison to the SCI control group. Furthermore, compared to PAME (50 mg/kg), treatment with methylprednisolone effectively ( *p* < 0.05) produced a significant increase in thermal hyperalgesia, mechano-tactile, locomotor activity, and MNCV ( Figure 2 ).

### Spinal IL and TNF-α protein levels

The SCI control rats showed a significant ( *p* < 0.05) increase in spinal IL and TNF-α levels compared to the sham and normal rats. The alterations in spinal IL and TNF-α levels were significantly ( *p* < 0.05) ameliorated by the PAME (100 and 200 mg/kg) intervention. Furthermore, when methylprednisolone was administered to the rats, a significant ( *p* < 0.05) effect was noted in terms of a decrease in IL and TNF-α levels in the spinal cord compared to the SCI control rats ( Table 2 ).

**Table 2:** Levels of inflammatory cytokines in the spinal cord

Parameter	Normal	Sham	SCI-control	MP (30 mg/kg)	PA (50 mg/kg)	PA (100 mg/kg)	PA (200 mg/kg)
TNF-α (pg/mL)	9.21 ± 1.12	18.96 ± 1.35	75.96 ± 0.96 ^#,&^	18.83 ± 1.23* ^,$^	70.08 ± 1.67	46.33 ± 1.06* ^,$^	35.46 ± 1.06* ^,$^
IL-1β (pg/mL)	79.76 ± 5.70	107.20 ± 4.54	258.50 ± 5.87 ^#,&^	116.50 ± 5.33* ^,$^	251.20 ± 3.28	176.20 ± 4.08* ^,$^	148.50 ± 5.25* ^,$^
IL-6 (pg/mL)	66.43 ± 3.50	63.97 ± 2.48	141.80 ± 3.38 ^#,&^	82.20 ± 2.29* ^,$^	137.10 ± 3.46	95.75 ± 3.41* ^,$^	78.75 ± 1.52* ^,$^

Abbreviations: IL, interleukin; MP, methylprednisolone; N, normal; PA, *Phyllanthus amarus* ; S, sham; SCI, spinal cord injury; TNF-α, tumor necrosis factor alpha.

Notes: Results are expressed as mean and standard error of the mean ( *n* = 6) values. The statistical analysis was conducted using one-way analysis of variance (ANOVA) with the Tukey's post-hoc test for multiple comparisons. *p* < 0.05: *SCI-control; ^#^ sham; ^&^ normal; and ^$^ one another (methylprednisolone versus *P. amarus* ).

### Spinal Bcl-2, Bax, and caspase-3 protein expressions

**Figure 3:**
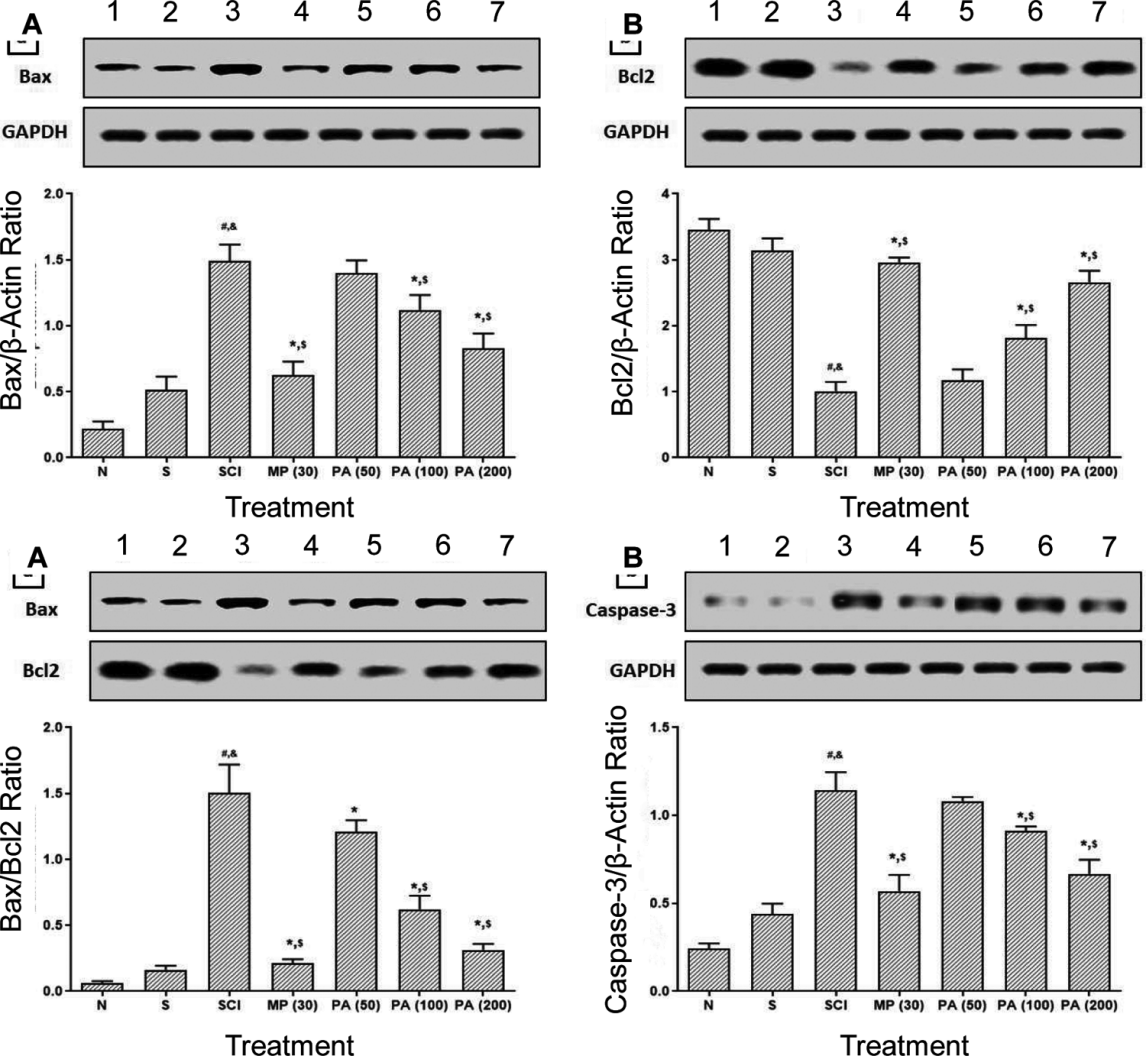
Abbreviations: Bax, BCL2-associated X protein; Bcl2, B-cell leukemia/lymphoma 2; MP, methylprednisolone; N, normal; PA, *Phyllanthus amarus* ; S, sham; SCI, spinal cord injury. Notes: Results are expressed as mean and standard error of the mean ( *n* = 6) values. The statistical analysis was conducted using one-way analysis of variance (ANOVA) with Tukey's post-hoc test for multiple comparisons. Protein expression of normal rat (Lane 1), sham rat (Lane 2), SCI-control rat (Lane 3), methylprednisolone-treated (30 mg/kg) rat (Lane 4), PA-treated (50 mg/kg) rat (Lane 5), PA-treated (100 mg/kg) rat (Lane 6) and PA-treated (200 mg/kg) rat (Lane 6). *p* < 0.05: *SCI-control; ^#^ sham; ^&^ normal; and ^$^ one another (methylprednisolone versus *P. amarus* ). Protein expressions of Bax ( **A** ), Bcl2 ( **B** ), Bax:Bcl2 ratio ( **C** ), and caspase-3 ( **D** ) in the spinal cord.

As demonstrated in Figure 3 , there was a significant upregulation ( *p* < 0.05) in the protein expression of caspase-3 and Bax in the spinal cord, whereas protein expression of Bcl-2 was significantly downregulated ( *p* < 0.05) in SCI-control rats compared to sham and normal rats. Intervention with PAME (100 and 200 mg/kg) and methylprednisolone significantly downregulated ( *p* < 0.05) spinal caspase-3 and Bax protein expression and upregulated protein expression of spinal Bcl-2 in the SCI control group. The SCI-induced alterations in spinal Bcl-2, Bax, and caspase-3 protein expressions were more significantly ( *p* < 0.05) inhibited in rats treated with methylprednisolone than in those treated with PAME (50 mg/kg).

### Spinal cord histology

**Figure 4:**
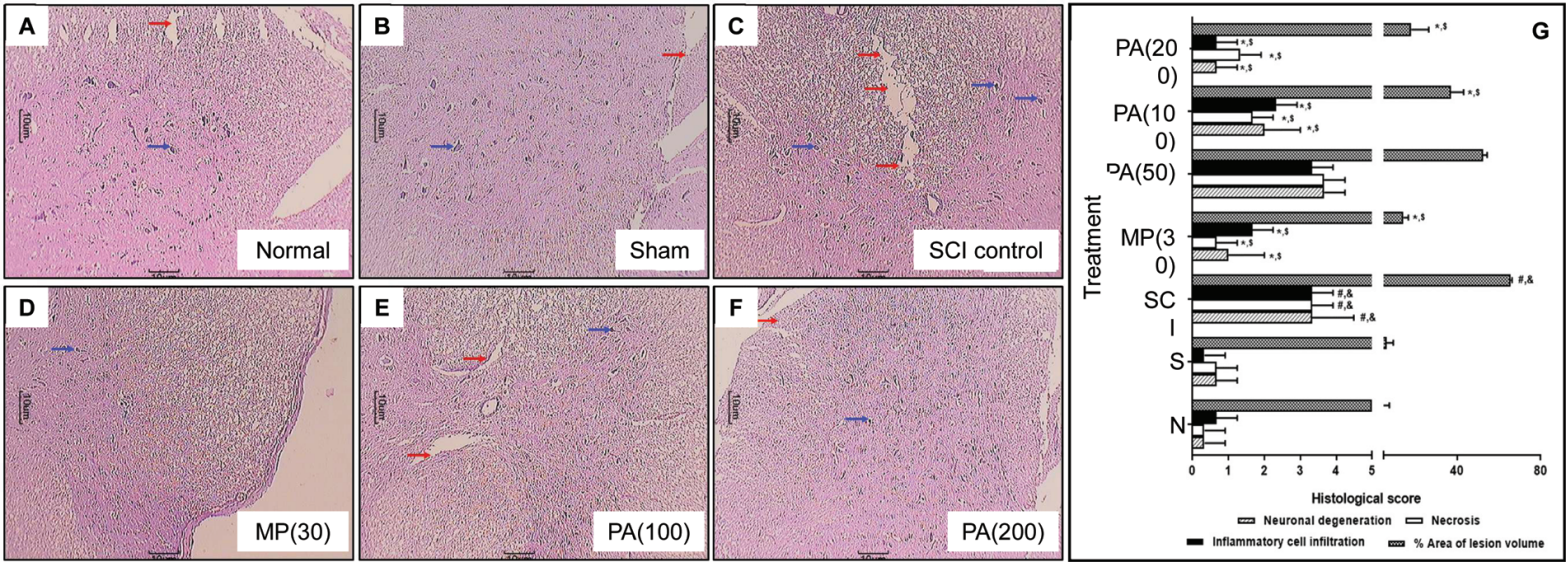
Abbreviations: MP, methylprednisolone; N, normal; PA, *Phyllanthus amarus* ; S, sham; SCI, spinal cord injury. Notes: Results are expressed as mean and standard error of the mean ( *n* = 6) values. The statistical analysis was conducted using one-way analysis of variance (ANOVA) with the Kruskal-Wallis post-hoc test for multiple comparisons. *p* < 0.05: *SCI-control; ^#^ sham; ^&^ normal; and ^$^ one another (methylprednisolone versus *Phyllanthus amarus* ). Alteration in spinal cord histology induced by SCI. Spinal cord microscopic images of a normal rat ( **A** ), sham rat ( **B** ), SCI-control rat ( **C** ), methylprednisolone-treated (30 mg/kg) rat ( **D** ), PA-treated (100 mg/kg) rat ( **E** ), and PA-treated (200 mg/kg) ( **F** ). Quantitative representation of *P. amarus* on SCI-induced alterations in rat spinal cord histology ( **G** ). Spinal cord sections stained with hematoxylin and eosin (H&E). Images at 40x of magnification are typical and representative of each study group. Inflammatory infiltration (blue arrow) and necrosis (red arrows).

Laminectomy at the T10 level caused aberrations in the spinal cord, as evidenced by alterations in the histological score, including a significant ( *p* < 0.05) increase in inflammatory nerve cell infiltration, neuronal degeneration, congestion, necrosis, and cell edema in SCI-control rats ( Figure 4C ) compared to sham and normal rats ( Figure 4A,B ). Spinal tissue from methylprednisolone-treated rats showed a significant reduction ( *p* < 0.05) in inflammatory infiltration, congestion, necrosis, and cell edema compared to SCI-control rats ( Figure 4D ). The PAME (100 and 200 mg/kg) treatment showed a protective effect, which was evident by a significant ( *p* < 0.05) reduction in inflammatory infiltration, neuronal degeneration, congestion, cell necrosis, and edema compared to SCI-control rats ( Figure 4E , F , G ).

## DISCUSSION

The current study investigated the potential of phyllanthin identified in *P. amarus* to ameliorate the behavioral, biochemical, molecular, and histological aberrations caused by SCI-induced neuronal damage in rodents.

Neuropathy of peripheral nerves is a frequent consequence of SCI that causes impairment of varying degrees in sensory, motor, and autonomic functions. 5 Nerve-conduction studies 12 have long been incorporated as diagnostic, staging, and prognostic tools to manage SCI. They determine how fast electrical impulses flow via the peripheral nerves. Abnormal consequences may be observed when an anomaly arises in the Ranvier axon, myelin, and nodes. 13 Due to structural changes such as the diminution of myelinated fibers and axonal atrophy, there is a reduction in MNCV. 14 Reduced MNCV, which is typically asymptomatic, is regarded as the first quantitative objective marker of polyneuropathy. 12 14 Induction of injury to the spinal cord produces considerable degradation in MNCV, resulting in motor fiber damage and in a reduction in paw withdrawal latency in rats. The present study revealed that pain thresholds in behavioral tests were significantly alleviated after PAME administration compared to the SCI-control rats. Our findings align with those of previous research ^15^ in whivh an alcoholic extract of *P. amarus* showed protective effects by preventing the deterioration of MNCV in diabetic rats.

Inflammation, a protective response to injury or infection, can become chronic, contributing to various disorders associated with pain. 16 Challenges in intervening with inflammatory pain currently stem from the constrained effectiveness of available analgesics, attributed to the need for personalized treatment strategies and the potential side effects, such as addiction, dependence, tolerance, gastrointestinal bleeding, renal impairment, and cardiovascular events. 17 Injury to the spinal cord triggers the activation of leukotrienes, bradykinin, TNF-α, ILs, and other cytokines and chemokines. 12 Abnormal levels of inflammatory mediators, including TNF-α, IL-1, IL-6, type-I and -II interferons, and IL-10, disrupt immune function and promote tissue inflammation, ultimately resulting in organ damage. 18 19 Tumor necrosis factor alpha is crucial in the cytokine cascade associated with numerous inflammatory diseases. Its involvement in the pathogenesis of autoimmune and inflammatory diseases suggests that it is a potential therapeutic target. 20 21 It plays a vital role in peripheral and central sensitization pathways, which are linked to the onset of inflammation during neuropathic pain. 22 23 The IL-1 family induces pain, inflammation, and autoimmune conditions, with IL-1β having strong proinflammatory effects on various cells, contributing to acute and chronic inflammation and pain. 24 Additionally, IL-6, a pleiotropic cytokine, is involved in inflammation, hematopoiesis, embryonic development, bone metabolism, and other immune responses. In the present study, SCI was linked to increased concentrations of ILs and TNF-α within the spinal cord tissue; however, the administration of PAME showed an effective reduction in ILs and TNF-α. Our findings are consistent with those of the literature, which show that *P. amarus* alcoholic extract reduces IL-6 and TNF-α levels, 25 suggesting its anti-inflammatory potential.

Understanding and modulating apoptosis in SCI is crucial to develop effective treatments and improve the outcomes of individuals with SCI. Programmed cell death, also known as apoptosis, is crucial to trigger and sustain SCI. Following trauma, apoptosis is triggered in neurons and glial cells, an initial response to the trauma. 26 The mechanical damage caused by the injury often leads to secondary damage, in which apoptosis further contributes to neuronal loss. 27 The loss of neurons can significantly impair functional recovery and contribute to long-term disability. The Bax is a pro-apoptotic protein that promotes apoptosis, whereas Bcl2 is an anti-apoptotic protein that inhibits apoptosis. 28 29 During SCI, Bax undergoes structural alteration, moves to the mitochondria, and facilitates cytochrome C release into the cytoplasmic space. This release activates caspases, leading to programmed cell death. 30 31 It is upregulated in response to secondary injuries, including inflammation and oxidative stress. Furthermore, initiator caspases are activated through Bax, which subsequently activates effector caspases that execute apoptosis. 32 33 In the present investigation, damage to the spinal cord resulted in upregulated expression of caspase-3 and Bax proteins in the spinal cord; however, the administration of PAME inhibited these proapoptotic proteins, suggesting the antiapoptotic potential of *P. amarus.* Recently, Afolabi et al. 34 (2022) documented that methanolic leaf extracts of *P. amarus* suppressed ischemia reperfusion-induced Bax/caspase 3 activation, suggesting its beneficial effect against intestinal and hepatic injuries. The results of the present study align with and support the conclusions drawn by earlier researchers. 34

Studies have reported the safety and efficacy of therapeutic moieties of herbal origin in managing SCI. 35 36 Researchers have documented the safety of *Phyllanthus* during the treatment of various maladies, including hepatitis B 37 38 and viral hepatitis. 39 40 Thus, *P. amarus* can be considered a promising treatment option for SCI.

In the current study, phyllanthin identified in *P. amarus* showed potential neuroprotective benefits against SCI by moderating impairments in behavioral (allodynia, hyperalgesia, and MNCV) parameters, elevated inflammatory mediators (IL-1β, IL-6, and TNF-α), and deactivating the apoptotic signaling (Bax/caspase-3) pathway (graphical abstract). Based on these findings, we can conclude that *Phyllanthus* can be considered a promising neuroprotective agent against SCI.
